# The association of preoperative anxiety and depression with neurocognitive disorder following oncological surgery

**DOI:** 10.1002/jso.25836

**Published:** 2020-01-12

**Authors:** Jing Du, Matthijs Plas, Anthony R. Absalom, Barbara L. van Leeuwen, Geertruida H. de Bock

**Affiliations:** ^1^ Department of Epidemiology University of Groningen, University Medical Center Groningen, Hanzeplein Groningen The Netherlands; ^2^ Department of Surgery University of Groningen, University Medical Center Groningen, Hanzeplein Groningen The Netherlands; ^3^ Department of Anesthesiology University of Groningen, University Medical Center Groningen, Hanzeplein Groningen The Netherlands

**Keywords:** cognitive dysfunction, depression, neoplasm, surgical procedure

## Abstract

**Background:**

The proposed underlying mechanisms of anxiety and depression, and of postoperative neurocognitive disorder (NCD), each include immune system involvement. Therefore, the aims of this study were to investigate the incidence of postoperative NCD 3 months after surgery among oncological patients undergoing surgery and to evaluate the role of preoperative anxiety and depression.

**Method:**

A consecutive series of patients (age ≥ 18 years) undergoing surgery for the removal of solid tumors were included (n = 218). Cognitive performance was assessed preoperatively and at 3 months postoperatively. Preoperative anxiety and depression were evaluated using the Hospital Anxiety and Depression Scale.

**Results:**

NCD affected 12.3% of elderly patients (age ≥ 70 years, n = 57) at 3 months after surgery, with executive function mostly affected. By contrast, 8.4% of younger patients (age < 70 years, n = 107) were affected, with information processing speed mostly affected. Low educational attainment was a risk factor (OR, 6.0; 95% CI, 1.9–19.0) of overall NCD, whereas preoperative anxiety was associated with decline in the domain of executive function.

**Conclusion:**

Postoperative NCD is a complication of oncological surgery for all adults instead of the elderly only. Preoperative anxiety was associated with an increased risk of executive function decline, and low educational attainment was a key factor for overall NCD.

## 1 INTRODUCTION

The ageing society has led to rapid increases in the number of patients with cancer across all ages.^1^ The combination of the rise in cancer incidence, and surgery as one of the main treatments for solid tumors results in the forecast that the number of oncological patients eligible for surgery will also increase.^2^ The treatment phase of oncological disease is accompanied by (symptoms of) anxiety and depression, where 19% and 12.9% of patients show signs of anxiety and depression.^3^ Undergoing surgery, fear of cancer recurrence and death, and the risk of potential adverse postoperative outcomes have influence on anxiety and depression and affect quality of life negatively.^4^ An adverse outcome after surgery considered particularly relevant in (but not restricted to) the elderly is postoperative cognitive decline.^5^ This deterioration in cognitive functioning following surgery has been termed as postoperative neurocognitive disorder (NCD) recently.^6^

Although it has been reported that postoperative NCD is a multifactorial phenomenon, there are few well‐established risk factors.^7^ Accumulating evidence suggests that postoperative NCD might result from increased inflammatory activity.^8^, ^9^ As both anxiety and depression are associated with increased inflammatory activity, preoperative symptoms might predispose patients for the development of postoperative NCD.^10^, ^11^ In literature most studies focused on the elderly, but this subject would be interesting for both the young and the old, as younger patients tend to experience more anxiety and depression during cancer treatment but older patients are at increased risk for postoperative NCD by advancing age.^12^, ^13^ Although definition, degree and duration of postoperative NCD are well explored, the influence of anxiety and depression on the development of postoperative NCD has not been investigated extensively.

We hypothesize that patients with (symptoms of) anxiety and depression have a higher risk for the development of postoperative NCD compared with patients without these symptoms. The aim of this study is to investigate the incidence of postoperative NCD 3 months after surgery among young and older patients undergoing surgery for cancer and to evaluate the role of preoperative (symptoms of) anxiety and depression.

## 2 METHODS

### 2.1 Study design

This study is embedded in the prospective observational study “PICNIC‐B‐HAPPY” (Predicting Postoperative Outcome in Elderly Surgical Cancer Patients: Biomarkers and Handgrip Strength as Predictors of Postoperative Outcome in the Elderly), conducted at the University Medical Center Groningen (UMCG, Groningen, The Netherlands) from August 2014 until March 2017. The study was registered on the Dutch Clinical Trial Database (trial number NL45602.042.14), following approval by the Medical Ethics Committee of the UMCG. A consecutive series of patients aged 18 years and over, admitted to the UMCG for surgical removal of a solid tumor (including gynecological tract, digestive tract, soft tissue) were invited to participate. Patients were excluded if surgery was scheduled in less than 24 hours after inclusion or if patients had any physical condition that could potentially impede compliance with the study, such as severe visual or auditory impairment, recent history of stroke or insufficient understanding of the Dutch language. Data collection was conducted in accordance with the Declaration of Helsinki.^14^ Written informed consent was obtained from all patients in accordance with local regulations, and patients’ identities were anonymized by coding data before statistical analysis.

### 2.2 Outcomes

The primary study outcome was the incidence of postoperative NCD 3 months after surgery in young and older patients undergoing surgery for cancer. Secondary study outcomes were the prevalence of preoperative (symptoms of) anxiety and depression in young and older patients undergoing surgery for cancer and the associations between risk factors, including preoperative anxiety and depression, with postoperative NCD 3 months after surgery.

### 2.3 Definitions and data collection

Neuropsychological tests to determine performance in three cognitive domains (memory, executive function and information processing speed) were conducted at baseline (approximately 2 weeks before surgery) and 3 months after surgery. The Dutch version of the Rey Auditory Verbal Learning Test (RAVLT) for immediate and delayed recall, the Trail Making Test part A (TMT‐A) and B (TMT‐B), and Ruff's Figural Fluency Test (RFFT) were used to determine neurocognitive performance in the domains of memory, executive function and information processing speed. The RAVLT was used as an indicator of memory and expressed as the total number of words correctly remembered during the five immediate recall trials (lowest score, 0; highest score, 75) and the total number of words remembered at the delayed recall trial (lowest score, 0; highest score, 15).^15^ The TMT‐A was used as an indicator of information processing speed and expressed as the number of seconds it took to complete the TMT‐A (lowest score, 0; highest score, 480).^16^ Performance on executive function was expressed as total number of the number of seconds it took to complete the TMT‐B (lowest score, 0; highest score, 480) and the unique designs drawn in parts 1 to 5 (lowest score, 0; highest score, 175) of the RFFT.^5^, ^16^, ^17^ A dedicated nurse and a medical or neuropsychology graduate student were trained on neuropsychological test administration and relevant interview techniques by a neuropsychologist. All measures were administered and scored in a standardized manner. Postoperative NCD was studied at overall and per cognitive domain. Overall postoperative NCD was defined as a ≥25% decline in the performance scores compared with the baseline score, in at least two of the five tests.^5^, ^18^ Whereas domain postoperative NCD was defined as a ≥25% decline in the performance scores in a specific domain compared with the baseline score in that domain.

Anxiety and depression symptoms were assessed using the Hospital Anxiety and Depression Scale (HADS) at baseline (approximately 2 weeks before surgery).^19^ The HADS is a 14‐item screening tool that focuses on nonphysical symptoms of anxiety and depression, using 7 items for anxiety (HADS‐A) and depression (HADS‐D), respectively. Responses are rated from 0 to 3 points, total scores on HADS‐A and HADS‐D may range from 0 to 21 points. Optimal balance between sensitivity and specificity for HADS as screening instrument is achieved most frequently at a cut‐off score of ≥8 for HADS‐A and HADS‐D. For both subscales sensitivities and specificities are approximately 0.80.^20^

Patient, psychosocial, disease, and treatment details were collected prospectively from baseline. Socioeconomic status (SES) was estimated for each patient, using an area‐based measure (postal codes) provided by the Dutch governmental organization *Sociaal Cultureel Planbureau* that assigned an overall score for income level, degree of unemployment and percentage of low education level. Accordingly, postal codes were assigned to 3 SES categories: low (fourth and fifth quintile), intermediate (third quintile), and high (first and second quintile). Independence was assessed using the Instrumental Activities of Daily Living (IADL) scale, and frailty was assessed using the Groningen Frailty Index (GFI).^21^, ^22^ Preoperative cognitive function was assessed using the Mini‐Mental State Examination (MMSE), whereas comorbidity was assessed using the Charlson Comorbidity Index.^23^, ^24^ Tumor stage was assessed using the TNM classification system, and anesthetic risk was estimated 24 hours before surgery using the American Society for An esthesiologist scale (ASA).^25^

Educational attainment was categorized into primary school or below, and higher than primary school (In the Netherlands, most children finish primary school at the age of 12). Socioeconomic status was categorized into low, intermediate and high.^26^ A surgical procedure with an anesthesia duration of >210 minutes was defined as major surgery.^5^ A history of chemotherapy or radiotherapy indicated either neoadjuvant or postoperative (within 3 months) therapy. Patients aged ≥70 years were considered as elderly and patients aged <70 years as young. Clinically relevant or literature‐based cut‐off scores were used to dichotomize the variables, as detailed in Appendix 1.

### 2.4 Data analysis and statistics

Patients with at least one complete cognitive test series (out of the maximum of five complete cognitive test series) were included in the analysis. A cognitive test series consists of baseline testing and testing at 3 months postoperatively. *Χ* ^2^ tests were performed to assess whether there were differences between included and excluded patients. Cognitive assessment scores are presented as medians and interquartile ranges (IQRs). Wilcoxon‐signed rank tests were used to assess changes in cognitive performance over time. Univariate and multivariate logistic regression analysis were performed to evaluate the associations between risk factors and NCD at 3 months after surgery. Variables with *P* values of <.15 in univariate analyses were included in the multivariable analyses. Given the specific interest in the role of preoperative depression, HADS‐D outcomes were retained in the multivariate models. A rule of at least five events per predictor variable in the multivariable analysis was applied.^27^ Odds ratios (ORs) and their corresponding 95% confidence intervals (95% CIs) were estimated and are reported. *P* values of <.05 were considered to indicate statistical significance. Data analysis was performed using IBM SPSS, Version 23 (IBM Corp, Armonk, NY) and and GraphPad Prism version 5.04 (GraphPad Software, San Diego, CA).

## 3 RESULTS

**Figure 1:**
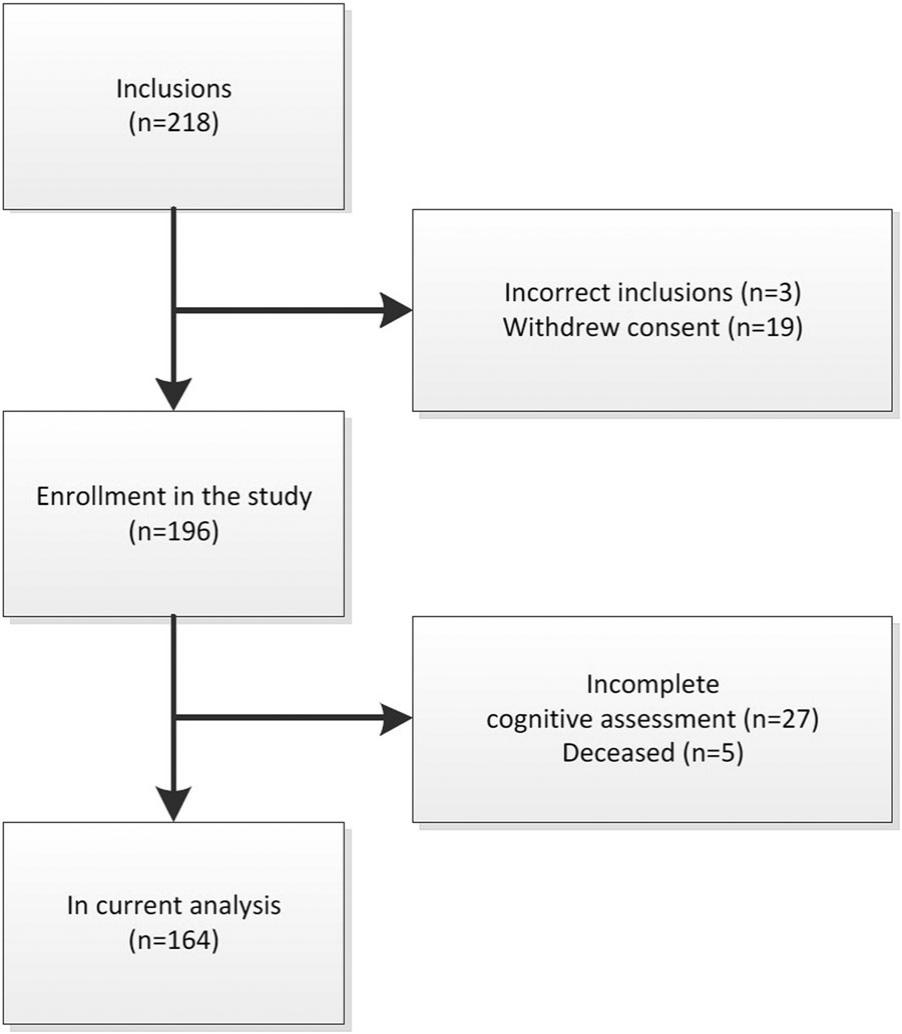
Flowchart from in‐ and excluded patients. Of the 218 included patients, 22 were excluded before surgery. Three months postoperatively 32 patients were excluded. The sample of current analysis included 164 patients

Figure 1 shows the flowchart of the patients included in the “PICNIC‐B‐HAPPY” study. Of the 218 consecutive patients enrolled in the study, 3 patients (1.4%) were excluded as they did not undergo surgery at the UMCG, and a further 19 patients (8.7%) withdrew their consent before surgery. In addition, 32 of the remaining 196 patients (16.3%) were excluded from the analysis due to no complete cognitive assessment data or death 3 months after surgery. Poor health status was the main reason leading to incomplete cognitive assessment data, especially at 3 months after surgery as patients were unable to undergo or finish the formal assessments. Data for the remaining 164 patients (75.2%) were analyzed in the current study.

Patients included and excluded from the analysis were comparable in health status (Table 1). Of the164 included patients, most had education to a level higher than primary school (87.1%), but also had low SES (72.5%) and high rates of comorbidities (73.8%). More than half of the included patients either had a body mass index exceeding 25 kg/m^2^ (63.8%) or underwent invasive surgery (69.5%). To the elderly group, 57 patients were assigned with a median age of 75 years (IQR, 72.5–78.5) and 107 patients to the young group with a median age of 61 years (IQR, 52‐67).

**Table 1:** Patient, psychosocial, disease, and treatment details (n = 218)

Risk factors	Included (n = 164)	Excluded* (n = 54)	*P* **
	% (n)	% (n)	
Patient and psychosocial characteristics			
Age (years)			.388
<70	65.2 (107)	58.1 (25)	
≥70	34.8 (57)	41.9 (18)	
Gender			.178
Female	46.3 (76)	34.9 (15)	
Male	53.7 (88)	65.1 (28)	
Educational level			.998
Primary school or lower	12.9 (21)	12.9 (4)	
Higher than primary school	87.1 (142)	87.1 (27)	
Social Economic Status^a^			.284
Low (7‐10)	72.5 (116)	64.8 (35)	
Intermediate or high (1‐6)	27.5 (44)	35.2 (19)	
Living situation			.762
Lives independently with others	76.7 (125)	74.3 (26)	
Lives alone	23.3 (38)	25.7 (9)	
Instrumental Activities of Daily Living^b^			.255
=8	83.9 (135)	75.0 (21)	
<8	16.1 (26)	25.0 (7)	
Body Mass Index^a^			.127
Normal (<25)	36.3 (58)	21.4 (6)	
Overweight (≥25)	63.8 (102)	78.6 (22)	
Groningen Frailty Indicator			.951
<4	79.3 (130)	78.8 (26)	
≥4	20.7 (34)	21.2 (7)	
Mini‐Mental State Examination			.245
≤26	6.1 (10)	12.9 (4)	
>26	93.9 (154)	87.1 (27)	
Charlson Comorbidity Index			.330
≤2	26.2 (43)	18.2 (6)	
>2	73.8 (121)	81.8 (27)	
Hospital Anxiety and Depression Scale—Anxiety^c^			.327
No (≤7)	84.6 (137)	77.4 (24)	
Mild or moderate (8‐14)	15.4 (25)	22.6 (7)	
Hospital Anxiety and Depression Scale—Depression^c^			.769
No (≤7)	88.3 (143)	87.1 (27)	
Mild or moderate (8‐14)	11.7 (19)	12.9 (4)	
Disease and treatment characteristics			
Tumor stage^c^			.360
Benign, 0, I, or II	43.8 (71)	35.3 (12)	
III or IV	56.2 (91)	64.7 (22)	
American Society of Anesthesiologists physical status classification			.135
<3	79.9 (131)	67.7 (21)	
≥3	20.1 (33)	32.3 (10)	
Invasive surgery			.534
No	30.5 (50)	25.0 (8)	
Yes	69.5 (114)	75.0 (24)	
Major surgery			.204
No	40.9 (67)	53.3 (16)	
Yes	59.1 (97)	46.7 (14)	
History of chemo/radiotherapy			.189
No	54.8 (85)	41.9 (13)	
Yes	45.2 (70)	58.1 (18)	
PostoperatIve delirium			.657
No	94.5 (155)	92.6 (25)	
Yes	5.5 (9)	7.4 (2)	

* Patients were excluded if they had all the five tests incomplete or withdrew their informed consent. In the excluded group, basic characteristics were missing due to withdrawal of informed consent before the first assessment.

^**^ *P* values were derived from *χ* ^2^ tests or Fisher's exact tests. A surgical procedure with an anesthesia duration of > 210 min was defined as major surgery.

^a^ Four missing data

^b^ Three missing data

^c^ Two missing data

Preoperatively, 25 (15.4%) patients had mild or moderate signs of anxiety and 19 (11.7%) patients had mild or moderate signs of depression. In the elderly group 5 (9.0%) patients had mild or moderate signs of anxiety preoperatively compared with 20 (18.7%) patients in the younger group. Mild or moderate signs of depression preoperatively were seen in 7 (12.3%) patients in the elderly group in contrast to 12 (11.2%) patients in the younger group.

**Figure 2:**
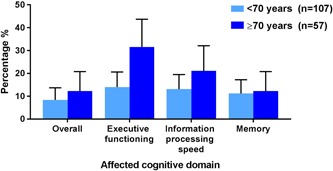
Cognitive decline at 3 months postoperatively. Data show the results of neurocognitive disorder overall and at the domain level as incidence (%) (95% confidence interval) [Color figure can be viewed at wileyonlinelibrary.com]

Table 2 shows the results of the neuropsychological tests at baseline and at 3 months postoperatively. Overall, there was statistically significant improvement in cognitive function in 87 patients (53.0% [95% CI, 45.4‐60.6]). However, 16 patients (9.8% [95% CI, 5.3‐14.4]) suffered from postoperative NCD at 3 months (Figure 2), including 7 in the elderly group (12.3% [95% CI, 3.8‐20.8]) and 9 in the young group (8.4% [95% CI, 3.1‐13.7]). In the elderly group, 31.6% (95% CI, 19.5‐43.7) encountered decline in the domain of executive function, while in the young group, 13.1% (95% CI, 6.7‐19.5) experienced decline in the domain of information processing speed. Incidences of neurocognitive change (disorder and improvement) from baseline to 3 months postoperatively are shown in Appendix 2.

**Table 2:** Cognitive assessment scores per test (n = 164)

Domain	Baseline median (interquartile range)	3 mo follow‐up median (interquartile range)	*P*
Memory			
RAVLT immediate recall	35.5 (26.0‐44.0)	41.0 (33.0‐52.0)	<.001
RAVLT delayed recall	7 (4‐9)	9 (6‐12)	<.001
Information processing speed			
TMT‐A	39.1 (29.8‐53.6)	36.8 (28.5‐50.1)	.021
Executive function			
RFFT	65.0 (47.5‐84.5)	78.0 (58.0‐100.0)	<.001
TMT‐B	92.7 (66.6‐128.8)	80.0 (60.1‐117.5)	.003

Abbreviations: RAVLT, Rey's Auditory Verbal Learning Test; RFFT, Ruff's Figural Fluency Test; TMT‐A, Trail Making Test part A; TMT‐B, Trail Making Test part B.

All *P* values were derived from Wilcoxon singed rank tests paired (*P* < .05 was considered significant).

The results of logistic regression analysis for overall postoperative NCD are shown in Table 3. Low education attainment (OR, 6.1 [95% CI, 1.4‐26.0]) was identified as a risk factor, while tumor stage III/IV (OR, 0.3 [95% CI, 0.1‐0.9]) was identified as an apparent protective factor of postoperative NCD. Despite the expectations, a preoperative HADS‐A or HADS‐D score > 7, was not associated with NCD 3 months after surgery.

**Table 3:** Logistic regression analysis for neurocognitive disorder by 3 mo postoperatively (n = 164)

	Univariable (n = 16)		Multivariable (n = 16)	
Risk factors	OR (95% CI)	*P*	OR (95% CI)	*P*
Patient and psychosocial characteristics				
Age, y		.397		
<70	1			
≥70	1.6 (0.6‐4.5)			
Gender		.754		
Female	1			
Male	1.2 (0.4‐3.3)			
Educational level^a^		**.003**		**.003**
Primary school or lower	**6.0 (1.9**‐**19.0)**		**6.0 (1.8**‐**20.1)**	
Higher than primary school	**1**		**1**	
Social Economic Status^d^		.073*		
Low (7‐10)	6.6 (0.8‐51.3)			
Intermediate or high (1‐6)	1			
Living situationa		.059*		
Lives alone	2.8 (1.0‐8.1)			
Lives independently with others	1			
Instrumental activities of daily living^c^		.790		
=8	1			
<8	0.8 (0.2‐3.8)			
Body mass index^d^		.277		
Normal (<25)	1			
Overweight (≥25)	0.6 (0.2‐1.6)			
Groningen frailty indicator		.875		
<4	1			
≥4	0.9 (0.2‐3.4)			
Mini‐mental state examination		.593		
≤26	1			
>26	1.8 (0.2‐16.7)			
Charlson Comorbidity Index		.880		
≤2	1			
>2	1.1 (0.3‐3.6)			
Hospital Anxiety and Depression Scale—Anxiety^b^		.262		
No (≤7)	1			
Mild or moderate (8‐14)	2.0 (0.6‐6.9)			
Hospital Anxiety and Depression Scale—Depression^b^		.891		.931
No (≤7)	1		1	
Mild or moderate (8‐14)	1.1 (0.2‐5.4)		1.1 (0.2‐5.7)	
Disease and treatment characteristics				
Tumor stage^b^		**.038**		**.041**
Benign, 0, I, or II	**1**		**1**	
III or IV	**0.3 (0.1**‐**0.9)**		**0.3 (0.1**‐**1.0)**	
American Society of Anesthesiologists physical status classification		.567		
< 3	1			
≥ 3	0.6 (0.1‐3.0)			
Invasive surgery		.971		
No	1			
Yes	1.0 (0.3‐3.1)			
Major surgery		.796		
No	1			
Yes	1.2 (0.4‐3.3)			
History of chemo/radiotherapy^e^		.983		
No	1			
Yes	1.0 (0.3‐2.8)			
Postopeartive delirium		n.a.		
No	1			
Yes	n.a.			

*Note*: Neurocognitive disorder was defined as a score drop of ≥25% on ≥2 of five tests. Depression and factors with a *P* value of <.15 in univariable analysis were included in the multivariable model. *P* values <.05 were considered significant. Bold values are considered statistically significant.

Abbreviations: CI, confidence interval; OR, odds ratio.

* Variables that were nonsignificant in a multivariable model.

^a^ One missing data.

^b^ Two missing data.

^c^ Three missing data.

^d^ Four missing data.

^e^ Nine missing data.

The risk factors for postoperative NCD at the domain level are shown in Table 4. For the memory domain, an ASA score ≥ 3 (OR, 3.7 [95% CI, 1.1‐12.5]) was identified as a risk factor, while tumor stage III/IV (OR, 0.3 [95% CI, 0.1‐0.9]) was identified as an apparent protective factor. In the executive function domain, age ≥ 70 (OR, 2.5 [95% CI, 1.1‐6.1]), education to primary school level or below (OR, 4.2 [95% CI, 1.5‐12.3]), and HADS‐A score > 7 (OR, 3.4 [95% CI, 1.1‐10.9]) were risk factors, while major surgery (OR, 0.3 [95% CI, 0.1‐0.7]) was a protective factor. Again, a preoperative HADS‐D score > 7 was not associated with postoperative NCD in specific domains.

**Table 4:** Logistic regression analysis for neurocognitive disorder per domain at 3 mo postoperatively (n = 164)

	Memory (n = 19)				Information processing speed (n = 26)				Executive function (n = 33)			
	Univariable		Multivariable		Univariable		Multivariable		Univariable		Multivariable	
Risk factors	OR (95% CI)	*P*	OR (95% CI)	*P*	OR (95% CI)	*P*	OR (95% CI)	*P*	OR (95% CI)	*P*	OR (95% CI)	*P*
Patient and psychosocial characteristics												
Age, y												
<70	1				1				1		1	
≥70	1.2 (0.4‐3.2)	.729			1.8 (0.8‐4.2)	.179			**2.8 (1.3**‐**6.1)**	**.010**	**2.5 (1.1**‐**6.1)**	**.037**
Gender												
Female	1				1				1			
Male	1.3 (0.5‐3.4)	.620			0.8 (0.4‐1.9)	.680			1.4 (0.6‐3.1)	.394		
Educational level^a^												
Primary school or lower	2.3 (0.7‐8.0)	.180			0.9 (0.2‐3.3)	.881			**4.7 (1.8**‐**12.3)**	**.002**	**4.2 (1.5**‐**12.3)**	**.008**
Higher than primary school	1				1				1		1	
Social Economic Status^d^												
Low (7‐10)	3.5 (0.8‐15.8)	.105*			1.0 (0.4‐2.5)	.923			2.4 (0.9‐6.7)	.091*		
Intermediate or high (1‐6)	1				1				1			
Living situationa												
Lives alone	1.3 (0.4‐3.8)	.680			0.7 (0.3‐2.1)	.568			2.3 (1.0‐5.2)	.054*		
Lives independently with others	1				1				1			
Instrumental Activities of Daily Living^c^												
=8	1				1				1			
<8	1.0 (0.3‐3.8)	.969			1.0 (0.3‐3.1)	.971			1.5 (0.6‐4.1)	.378		
Body Mass Index^d^												
Normal (<25)	1				1				1			
Overweight (≥25)	1.8 (0.6‐5.3)	.281			0.5 (0.2‐1.2)	.140*			0.7 (0.3‐1.6)	.409		
Groningen Frailty Indicator												
<4	1				1				1			
≥4	1.5 (0.5‐4.4)	.506			0.6 (0.2‐2.0)	.447			1.9 (0.8‐4.6)	.139*		
Mini‐Mental State Examination												
≤26	1				1				1			
>26	1.5 (0.2‐13.3)	.733			0.6 (0.1‐5.3)	.680			**5.6 (1.4**‐**22.3)**	**.014** *		
Charlson Comorbidity Index												
≤2	1				1				1			
>2	3.4 (0.7‐15.3)	.114*			1.0 (0.4‐2.5)	.962			1.8 (0.7‐4.7)	.236		
Hospital Anxiety and Depression Scale—Anxiety^b^												
No (≤7)	1				1				1		1	
Mild or moderate (8‐14)	1.6 (0.5‐5.2)	.468			1.0 (0.3‐3.2)	.982			2.1 (0.8‐5.4)	.122	**3.4 (1.1**‐**10.9)**	**.035**
Hospital Anxiety and Depression Scale—Depression^b^												
No (≤7)	1		1		1				1			
Mild or moderate (8‐14)	1.5 (0.4‐5.9)	.538	1.5 (0.4‐6.7)	0.559	0.6 (0.1‐2.7)	.483			2.0 (0.7‐5.7)	.203	1.2 (0.3‐4.3)	.745
Disease and treatment characteristics												
Tumor stage^b^												
Benign, 0, I, or II	1		1		1				1			
III or IV	**0.3 (0.1**‐**0.9)**	**.027**	**0.3 (0.1**‐**0.9)**	**0.035**	0.8 (0.3‐1.8)	.529			1.1 (0.5‐2.4)	.828		
American Society of Anesthesiologists physical status classification												
<3	1		1		1				1			
≥3	**4.3 (1.5**‐**11.9)**	**.006**	**4.1 (1.4**‐**11.8)**	**0.009**	0.1 (0.02‐1.0)	.055*			1.4 (0.6‐3.5)	.457		
Invasive surgery												
No	1				1				1			
Yes	2.7 (0.7‐9.7)	.130*			1.3 (0.5‐3.2)	.635			1.0 (0.4‐2.3)	.959		
Major surgery												
No	1				1				1		1	
Yes	2.8 (0.9‐9.0)	.077*			1.4 (0.6‐3.4)	.447			**0.4 (0.2**‐**0.8)**	**.013**	**0.3 (0.1**‐**0.7)**	**.008**
History of chemo/radiotherapy^e^												
No	1				1				1			
Yes	1.8 (0.7‐4.8)	.241			1.3 (0.6‐3.0)	.532			0.9 (0.4‐1.9)	.719		
Postopeartive delirium												
No	1				1				1			
Yes	2.6 (0.5‐13.8)	.271			0.64 (0.1‐5.3)	.680			1.1 (0.2‐5.7)	.879		

*Note*: Decline in a cognitive domain was defined as a decline of ≥25% on ≥1 test in that specific domain. Depression and factors with a *P* value of <.15 in the univariable analysis were included in a multivariable model. *P* values <.05 were considered significant. Bold values in the table indicate those considered statistically significant.

Abbreviations: CI, confidence interval; OR, odds ratio.

^a^ One missing data.

^b^ Two missing data.

^c^ Three missing data.

^d^ Four missing data.

^e^ Nine missing data.

* These variables were nonsignificant in a multivariable model.

## 4 DISCUSSION

In this study, 12% of patients aged ≥ 70 experienced NCD at 3 months after surgery, compared with 8% of those in patients aged < 70. Their affected domains were different in each group, with executive functioning most frequently affected in the elderly group (32%) and information processing speed most frequently affected in the young group (13%). Patients with lower educational attainment were at greater risk of postoperative NCD than those with higher educational attainment, whereas preoperative self‐reported anxiety was associated with decline at executive function domain.

### 4.1 Incidence of postoperative NCD and the domains most commonly affected

The finding that 12% of patients experienced postoperative NCD in the elderly group is consistent with the results of previous studies in elderly populations, which have shown that the incidence of NCD varies from 9.9% to 16% after noncardiac surgery.^5^, ^28^, ^29^ Only two studies have investigated the incidence of NCD in young adults (age < 65 years), and these reported incidences of 5.7% and 6.4%.^7^, ^13^ The slightly higher incidence in our study might reflect the slightly older population, the longer mean anaesthesia duration, and relatively invasive surgical procedures, which has been associated with a higher risk of NCD.^7^, ^13^ Furthermore, the differences in neuropsychological tests, the definitions of NCD and the study populations themselves might have affected the incidence of postoperative NCD.

The domain most vulnerable to decline was executive function, while memory function was least affected. This is consistent with previous findings among elderly patients with cancer.^5^ This distinction between cognitive domains supports the hypothesis that specific brain areas might respond differently to the perioperative inflammatory response.^30^ It was also notable that the incidence of decline in executive function was twice as high in the elderly group than in the young group. This might be due to the increased susceptibility and reactivity to inflammatory mediators of the areas associated with executive function in the aged brain, which in turn, exacerbated the neuroinflammatory response.^31^, ^32^

### 4.2 Preoperative (symptoms of) anxiety and depression

Preoperative anxiety was a risk factor of decline in executive function, controversially to what was hypothesized, preoperative depression was not associated with NCD 3 months after surgery in our adult population with cancer. It is possible that patients with symptoms of depression were less motivated to participate in the cognitive assessment in this study, thereby confounding the results. Supporting this theory, the incidence of self‐reported depression was only 11.6%, which is much lower than the reported 27% in a meta‐analysis of data for patients screened by self‐report instruments during treatment for cancer.^33^ The prevalence (15.2%) of anxiety in patients preoperatively in the current study lays in line with literature as on average 19% of patients show levels of anxiety in the clinical range during oncological treatment.^3^ However, younger patients experienced more (symptoms of) anxiety when compared with older patients. Studies point out that age is inversely related to emotional distress, and that younger patients tend to experience higher levels of anxiety due to a larger disruption of social and familial roles by diagnosis and treatment.^34^ Besides, younger patients have more limited life experience to help them cope with such traumatic situations.

### 4.3 Other risk factors

Educational attainment was found to be strongly associated with postoperative NCD. This is supported by a review that showed low educational attainment to be associated with an increased risk of NCD after surgery.^7^ It has been suggested that low education attainment itself might indicate lower cognitive reserves. That is, patients with high cognitive reserves may be better able to cope with disruptions by having more efficient and flexible cognitions than their peers with low reserves. Therefore, low education attainment might be a confounder rather than a risk factor of NCD.^35^ This might be expected when comparing NCD with a control group (eg, based on a *z*‐score cut‐off of 1.96).^13^, ^28^, ^36^ However, in our study, cognitive function was compared before and after surgery in the same group, where education attainment was unchanged.

It was notable that advanced tumor stage was protective against overall NCD and a decline in the memory domain. This may be because patients with advanced tumors had been physically ill and worried about their diagnosis and the upcoming surgery, potentially resulting in a higher chance of missing data and a worse performance on the preoperative neuropsychological tests.^37^ Those who underwent successful surgery might then have benefitted from physical improvement and stress relief that improved their postoperative cognitive performance. Equally, those who had continuing illness after surgery may have been unable to complete the cognitive assessment, as was observed in the International Study of Postoperative Cognitive Dysfunction in which continuing ill health after surgery commonly led to study withdrawal.^28^ Therefore, for patients with advanced tumors, improvements in cognitive performance scores might be expected in the research setting that are not seen in daily clinical practice.^38^

### 4.4 Strengths and limitations

There are several strengths of this study. First, the cohort was prospectively designed, and a research team was trained to conduct the tests in a standardized manner to avoid subjective bias in the delivery of the neuropsychological tests. Second, patient needs were prioritized when conducting the study, aiming to achieve a consecutive series of patients and to minimize dropout. When an additional visit to the hospital was a burden, assessments were completed at patients’ places of residence. Third, our deep investigation of NCD at the domain level in a wide age group, and not merely among the elderly, contributes to a greater understanding of the incidence of postoperative NCD and the domains of cognition that are affected. However, certain limitations of the present study should also be noted. The study was conducted in a tertiary referral center, which introduced selection bias. Patients referred to this hospital generally undergo more complex surgical procedures compared with the wider population who undergo surgery for cancer. In the study cohort, individuals with relatively worse health statuses also had a higher chance of being excluded. Meanwhile, patients with symptoms of depression were less motivated to participate which might limit the ability to assess the association between depression and postoperative NCD. There were 12% of the included patients unable to complete the follow‐up cognitive assessments at 3 months, and this dropout rate is comparable to that in other studies on this topic.^13^, ^39^ However, given that the excluded patients had a relatively worse health status and given that patients with impaired cognitive statuses are more likely to be lost to follow‐up, there is good reason to believe that the true incidence of NCD was even higher than that reported. It should also be noted that the failure to include a healthy control group prevented from accounting for a learning effect and might blur the true effect of surgery on cognitive change over time. However, a learning effect should cause postoperative cognitive performance to improve from baseline, reducing the chance of detecting NCD. This is yet another factor indicating that the true incidence of postoperative NCD could be even higher than we estimated.

### 4.5 Clinical implications and future perspectives

Clinicians and family members need to be aware of this increased vulnerability among patients with low educational attainment and preoperative anxiety symptoms and must be more vigilant for NCD in this population. Given that postoperative NCD also appears to occur at a high incidence among younger adult patients, researchers should investigate this phenomenon among patients of all ages in the future. A larger patient cohort from primary or secondary care will be needed to study the effect of psychosocial factors, specifically preoperative depression, on postoperative outcomes in the future. For a better understanding of pathophysiology, associations with inflammatory mediators, preoperative anxiety and depression and postoperative NCD should be explored, as the proposed underlying mechanisms involve the immune system.

## CONFLICT OF INTERESTS

The authors declare that there are no conflict of interests.

## AUTHOR CONTRIBUTIONS

JD cleaned the data, performed the statistical analysis and wrote the manuscript. MP participated in data collection and reviewed the manuscript. ARA designed the study and reviewed the manuscript. BLvL initiated the data collection, designed the study, and reviewed the manuscript. GHdB designed the study, supervised the statistical analysis, helped with the data interpretation, and reviewed the manuscript. All authors critically revised the initial draft of the manuscript and subsequent revisions. All authors approved the manuscript in its current form.

## ETHICS STATEMENT

The medical ethics committee of the University Medical Center Groningen (UMCG) approved this study, and all participants gave written informed consent. The study was performed in accordance with the Declaration of Helsinki.

## SYNOPSIS

A prospective study shows that postoperative NCD is a complication of oncological surgery for all adults instead of the elderly only. Preoperative anxiety is associated with an increased risk of executive function decline, and low educational attainment is a key factor for overall NCD.

## Data Availability

The data that support the findings of this study are available from the corresponding author upon reasonable request.

## APPENDIX 1 COMPONENTS OF THE PICNIC‐B‐HAPPY STUDY

**Table:**

Test	Short name.	Purpose	Cut‐off value for adverse results	Score Range
Socioeconomic status	SES	A combined score estimated for each four‐digit postal code area, based on income level, degree of unemployment and percentage of low education level by the “Sociaal Cultureel Planbureau”	High (first and second quintile); Intermediate (third quintile); Low (fourth and fifth quintile)	
Groningen Frailty Indicator	GFI	A 15‐item screening instrument measures the loss of functions and resources in four domains: physical, cognitive, social, and psychological to determine the level of frailty	≥4	0‐15
Instrumental activities of daily living	IADL	A questionnaire regarding eight items needed to perform independently to maintain independence in the community	<8	0‐8
Body mass index	BMI	A measure of body fat based on height and weight	≥25	N.A.
Hospital Anxiety and Depression Scale—Anxiety	HADS‐A	A questionnaire using seven items to identify anxiety	>7	0‐21
Hospital Anxiety and Depression Scale—Depression	HADS‐D	A questionnaire using seven items to identify depression	>7	0‐21
Mini Mental State Examination	MMSE	A test consisting of 11 questions to assess cognitive function	≤26	0‐30
Charlson Comorbidity Index	CCI	A scale predicts the 1‐year mortality for a patient who may have a range of comorbid conditions	≥2	1, 2, 3, or 6 for each condition
American Society for Anesthesiologist scale	ASA	To quantify preoperative physical status and estimate anaesthetic risk	≥3	1‐5

PICNIC‐B‐Happy study, Predicting postoperative outcome in elderly surgical cancer patients: Biomarkers and handgrip strength as predictors of postoperative outcome in the elderly.

## APPENDIX 2 INCIDENCE OF NEUROCOGNITIVE CHANGE FROM BASELINE TO 3 MONTHS POSTOPERATIVELY

**Table:**

	All (n = 158)		Elderly group (age ≥ 65 y; n = 89)		Young group (Age < 65 y; n = 69)	
	n	Incidence (%) (95% CI)	n	Incidence (%) (95% CI)	n	Incidence (%) (95% CI)
Neurocognitive disorder						
Overall	16	10.1 (5.9‐15.9)	10	11.2 (5.5‐19.7)	6	8.7 (3.3‐18.0)
Memory	17	10.8 (6.4‐16.7)	8	9.0 (4.0‐16.9)	9	13.0 (6.1‐23.3)
Information processing speed	26	16.5 (11.0‐23.2)	15	16.9 (9.8‐26.3)	11	15.9 (8.2‐26.7)
Executive function	30	19.0 (13.2‐26.0)	22	24.7 (16.2‐35.0)	8	11.6 (5.1‐21.6)
Neurocognitive improvement						
Overall	86	54.4 (46.3‐62.4)	54	60.7 (49.8‐70.9)	32	46.4 (34.3‐58.8)
Memory	93	58.9 (50.8‐66.6)	57	64.0 (53.2‐73.9)	36	52.2 (39.8‐64.4)
Information processing speed	35	22.2 (15.9‐29.4)	18	20.2 (12.4‐30.1)	17	24.6 (15.1‐36.5)
Executive function	82	51.9 (43.8‐59.9)	45	50.6 (39.8‐61.3)	37	53.6 (41.2‐65.7)

Postoperative neurocognitive disorder/improvement was defined as a postoperative disorder/improvement of ≥25% on ≥2 of 5 tests compared with the preoperative baseline assessment. Disorder/improvement on a specific domain was defined as a decline/improvement of ≥25% on ≥1 test in that specific domain.
